# Theories, determinants, and intervention models and approaches on inequalities of undernutrition amongst under fives: A literature review

**DOI:** 10.1002/hsr2.2078

**Published:** 2024-04-29

**Authors:** Kehinde Kazeem Kanmodi, Jimoh Amzat, Kafayat Aminu

**Affiliations:** ^1^ School of Dentistry University of Rwanda Kigali Rwanda; ^2^ Child Health and Wellbeing (CHAW) Program Cephas Health Research Initiative Inc Ibadan Nigeria; ^3^ Faculty of Dentistry University of Puthisastra Phnom Penh Cambodia; ^4^ School of Health and Life Sciences Teesside University Middlesbrough UK; ^5^ Department of Sociology Usmanu Danfodiyo University Sokoto Nigeria; ^6^ Center for Child and Adolescent Mental Health University College Hospital Ibadan Nigeria

**Keywords:** approach, children, determinants, global health, inequality, intervention, model, review, theory, undernutrition

## Abstract

**Background and Aims:**

One of the greatest public health problems of the 21st century is undernutrition in children under the age of 5 years (CAUFY). Globally, over 232 million CUAFY are undernourished and approximately 45% of mortality in this population are undernutrition‐induced. This paper reviewed and critically explained the factors perpetuating undernutrition in CUAFY in the global space. It further explained the multi‐level determinants that influence health inequalities and consequently exacerbate undernutrition amongst CUAFY globally. It also went further to explain the intervention models and approaches that can be used to tackle undernutrition in CUAFY.

**Methods/Literature Search Strategy:**

Demiris et al.'s approach to narrative review was utilized for this paper. Relevant articles on child nutrition were retrieved from multiple credible databases and websites of foremost health organizations. Using an iterative process, multiple combinations of search terms were done by stringing relevant key terms and their synonyms with Boolean Operators. This process was constantly refined to align search results with the study aim. Database search produced relevant and resourceful publications which were utilized to develop this review.

**Results:**

The global burden of undernutrition remains high, especially in Oceania with the highest prevalence of stunting and wasting (41.4% and 12.5%), with Africa and Asia following closely. Malnutrition eradication is a global health issue of high priority as demonstrated by the “Goal 2” of the Sustainable Development Goals (SDGs), and the United Nations (UN) Decade of Action on Nutrition 2016–2025. The review identified no significant positive outcome from previous interventions due to the endemic health inequalities. Determinants of the multi‐level health inequalities associated with undernutrition in CUAFY, and probable solutions are explained with theoretical models of health inequalities. A diagonal intervention approach was proposed as a viable solution to ending undernutrition in CUAFY.

**Conclusion:**

The application of relevant theoretical models and context‐specific intervention approaches can be utilized by stakeholders to close the existing inequality gaps, thereby reducing undernutrition amongst CUAFY globally.

## 1 INTRODUCTION

Undernutrition, also known as “poor nutrition,” refers to an insufficient intake of energy and nutrients necessary to meet an individual's nutritional requirements for maintaining good health.^1^, ^2^ According to the World Health Organization (WHO), undernutrition in children under the age of 5 years (CUAFY) manifests in four main forms: vitamin and mineral deficiencies, underweight, wasting (low weight‐for‐height), and stunting (low height‐for‐age).^3^ However, unlike underweight and micronutrient deficiencies, wasting and stunting have consistently been the forms of undernutrition among CUAFY reported globally over the past two decades (2000–2020).^4^, ^5^, ^6^

Undernutrition is a major cause of multiple and devastating morbidities in CAUFY. Common examples of these morbidities include low birth weight,^7^ congenital impairments,^8^ oral diseases,^9^ and developmental mental disorders.^8^, ^10^, ^11^

Undernutrition in CUAFY remains one of the greatest public health problems of the 21st century.^3^ Persistently, and globally, it has been responsible for numerous devastating long‐term effects on the public health and economic systems, some of which include long‐term economic growth retardation, the perpetuation of poverty cycle, reduction in national productivity, and an increase in the cost of healthcare.^3^ Pertinently, over 232 million CUAFY are undernourished globally and about 45% of deaths in CUAFY are due to undernutrition.^3^ The estimated impact of undernutrition and other forms of malnutrition is about US $500 per individual, costing all nations about US $3.5 trillion annually.^12^

Globally, undernutrition in CUAFY is perpetuated by multidimensional inequalities. This paper seek to provide a comprehensive review the global burden of undernutrition in CUAFY and draws on a range of public health theories on inequalities to critically explain the factors perpetuating undernutrition in CUAFY in the global space. It goes further to explain the multi‐level determinants that influence health inequalities which exacerbate undernutrition amongst CUAFY globally. This paper concludes with a discussion of theoretical models and approaches that can be used to close the existing inequality gaps to reduce undernutrition amongst CUAFY globally.

This narrative review represents a significant contribution to the existing literature, as the synthesis of scientific publications alongside a review of global‐level evidence on malnutrition in CAUFY is relatively uncommon. A literature search indicates that country‐level studies reporting the prevalence of undernutrition and the nutritional status of CUAFY, often as components of demographic and health surveys,^13^, ^14^ as well as reports on the global burden of malnutrition,^15^ are widespread. Empirical investigations on undernutrition in this demographic group are also prevalent, particularly in severely affected regions such as Sub‐Saharan Africa,^16^, ^17^, ^18^, ^19^ Asia,^20^ and low‐ and middle‐income countries in general.^21^

Furthermore, studies examining risk factors^14^, ^22^ and determinants of undernutrition,^23^, ^24^ predictors,^25^ and interventions aimed at addressing undernutrition among CUAFY^26^ are abundant. Existing literature has predominantly focused on prevalence, trends, determinants,^27^, ^28^ burden,^29^ and associated factors.^30^

Moreover, undernutrition in CUAFY is perpetuated by multidimensional inequalities, underscoring the necessity for a comprehensive understanding of the factors driving this issue. While existing literature acknowledges the complexity of these inequalities on a national scale,^31^, ^32^ there remains a gap in knowledge concerning the precise mechanisms exacerbating undernutrition among CUAFY globally. Specifically, there is a dearth of comprehensive theoretical analyses integrating public health theories on inequalities to elucidate the multi‐level determinants influencing health disparities in undernourished CUAFY populations worldwide.

To address this gap, this paper reviewed the global burden of undernutrition in CUAFY and critically examined the factors perpetuating this issue through the lens of public health theories on inequalities. By synthesizing existing literature and theoretical perspectives, this paper elucidated the complex interplay between social, economic, and environmental determinants of undernutrition among CUAFY. Furthermore, it highlighted theoretical models and approaches that can be leveraged to address existing inequality gaps and reduce undernutrition among CUAFY globally. By addressing this knowledge gap and proposing theoretical frameworks for intervention, this paper has contributed to the development of more effective strategies for combating undernutrition among CUAFY on a global scale.

## 2 LITERATURE SEARCH STRATEGY

In this review, we adopted the four steps approach to conducting a narrative review as Demiris et al. proposed.^33^ First, online searches of multiple databases (PubMed, CINAHL, SCOPUS, Web of Science, EBSCO, APA PsycINFO, Google Scholar, AJOL, and Embase) and websites of leading health organizations (including the WHO, United Nations (UN) Children Emergency Fund, and World Bank) were made to gather relevant literature on child nutrition. These databases and web sources were carefully chosen because they are leading and highly credible sources of literature in the medical and health science field.^34^, ^35^

Second, keywords were identified from the initial database search. Additional database search was done iteratively using Boolean operators (“AND” and “OR”) and the multiple combinations of the following keywords and search terms: “child,” “nutrition,” “undernutrition,” “malnutrition,” “stunting,” “underweight,” “wasting,” “micronutrient,” “inequality,” “determinants,” “prevention”, “strategies,” “interventions,” and “policies.” These keywords and synonyms were selected for the literature search strategy after a meticulous review of several terms pertaining to child undernutrition, inequalities, and interventions in the medical subject heading (MeSH) dictionary and Thesaurus.

Third, from the search, we identified relevant articles, reviews, book chapters, books, policy documents, gray literature, datasets, and websites containing information that were resourceful for this review. Then abstracts and full texts of the selected articles were screened for relevant information related to the subject matter. Lastly, we synthesized and summarized the findings reported in the identified literature, and these were used to develop the body of this narrative review.

## 3 OVERVIEW OF CURRENT GLOBAL BURDEN OF UNDERNUTRITION IN CUAFY

The recently released UNICEF/WHO/World Bank joint child malnutrition (global and regional) data, for the year 2020, on malnutrition amongst CUAFY revealed that about 22.0% and 6.7% of the global population of CUAFY are stunted and wasted, respectively.^36^ Although the joint data did not provide information on the burden of underweight, and vitamins and minerals deficiency in CUAFY, which is a limitation to the data, nonetheless, the UNICEF/WHO/World Bank joint child malnutrition data can still be regarded as the most authoritative data on the global and regional prevalence of undernutrition amongst CUAFY as the data is expected to have been collected through organized and coordinated efforts.

Furthermore, the studied regions, as depicted in the UNICEF/WHO/World Bank joint data were: Africa, Asia, Europe, Latin America and the Caribbean, Oceania (excluding Australia and New Zealand), Australia and New Zealand, and Northern America. In Africa, about 30.7% and 7.5% of CUAFY were stunted and wasted, respectively.^36^ In Asia, about 21.8%, and 11.8% of CUAFY were stunted and wasted, respectively.^36^ In Europe, about 4.5% of CUAFY were stunted^36^; with no data on wasted CUAFY in the region. In Latin America and the Caribbean, about 11.3% and 1.6% of CUAFY were stunted and wasted, respectively.^36^ In Oceania (excluding Australia and New Zealand), about 41.4% and 12.5% of CUAFY were stunted and wasted, respectively.^36^ In Australia and New Zealand, about 2.3% of CUAFY were stunted; with no data on wasted CUAFY in the region.^36^ In Northern America, 3.2% and 0.2% of CUAFY were stunted and wasted, respectively.^36^

The current distribution of the burden of undernutrition across the world regions, as described above, is skewed; this consolidates undernutrition in CUAFY as an enormous public health problem worsened by significant health inequalities.^36^ Oceania (excluding Australia and New Zealand) had the highest prevalence of stunting and wasting globally while, on the other hand, Northern America had the lowest prevalence of stunting and wasting.^36^ From the comparisons of the burden of stunting and wasting across regions, it can be inferred, although with caution, that stunting is the most predominant form of undernutrition among CUAFY globally, since there exists no global data on underweight and vitamins and minerals deficiency in CUAFY.

## 4 GLOBAL ERADICATION OF UNDERNUTRITION IN CUAFY: WHERE WE ARE

Due to the persistently high and devastating burden of malnutrition (particularly undernutrition) across the world regions, all the member states of the UN, in 2015, made a goal‐oriented declaration to eradicate food hunger, improve the nutritional status of people, support sustainable farming, and attain food security at the global level before the end of the year 2030.^37^ This goal is called “Goal 2” (second goal) of the 17 Sustainable Development Goals (SDGs) declared by all UN member States.^37^ The presence of Goal 2 in the list of 17 SDGs shows that malnutrition eradication is a global health issue of high priority.

Shortly after the SDGs declaration, the UN Generally Assembly also declared a 10‐year (2016 to 2025) period of action to eradicate malnutrition globally; this declaration was named the “United Nations Decade of Action on Nutrition 2016–2025.”^3^, ^38^ According to the UNSCN, the rationale behind the UN's declaration of an uninterrupted 10‐year combative action against the global malnutrition crisis was to strengthen already existing efforts toward undernutrition and micro‐nutrient deficiencies eradication which had been progressing too slowly and unevenly across different population groups, countries, and regions of the world.^38^

However, from 2015, when the UN declared its SDGs (of which global undernutrition eradication among CUAFY is an integral component of Goal 2 of the SDGs), till now, the public health efforts that had been channeled toward global undernutrition eradication, especially among CUAFY has not yielded the expected outcomes to a satisfactory level.^4^, ^5^ A comparative analysis of the 2015 and 2020 Global Nutrition Report reveals no significant reduction in the number of people being affected by undernutrition 5 years (2015–2020) after the UN SDGs declaration.^4^, ^5^ In 2015 alone, about 161 million and 51 million CUAFY were stunted and wasted, respectively.^5^ Meanwhile, for the year 2020, about 149 million and 45 million CUAFY were still stunted and wasted, respectively.^3^ Based on this trend, the global population of undernourished CUAFY had only reduced by 8.9% within the space of 5 years. The UN target was to eradicate malnutrition globally on or before 2030^38^; based on the current rate at which the prevalence of undernourished CUAFY is being brought down, it can be asserted confidently that undernutrition may not be eradicated among CUAFY within the next decade. Most probably, undernutrition will continue to linger as a public health problem beyond 2030 which may be attributed to the impact of the COVID‐19 pandemic and multiple humanitarian crises across different parts of the world.

## 5 SOCIAL GRADIENT AND THEORIES OF INEQUALITIES OF UNDERNUTRITION IN CUAFY

Undernutrition in CUAFY has not been eradicated globally due to associated health inequalities.^4^, ^39^ By way of definition, global health inequality refers to “health disparities, within and between countries, that are judged to be unfair, unjust, avoidable, and unnecessary (meaning: are neither inevitable nor un‐remediable) and that systematically burden populations rendered vulnerable by underlying social structures and political, economic, and legal institutions.”^40^ In other words, health inequality refers to “the systematic differences between more and less advantaged groups”^41^ (cited in McCartney et al.^39^). The above definitions show that health inequality is a multidimensional public health problem.^42^

**Figure 1:**
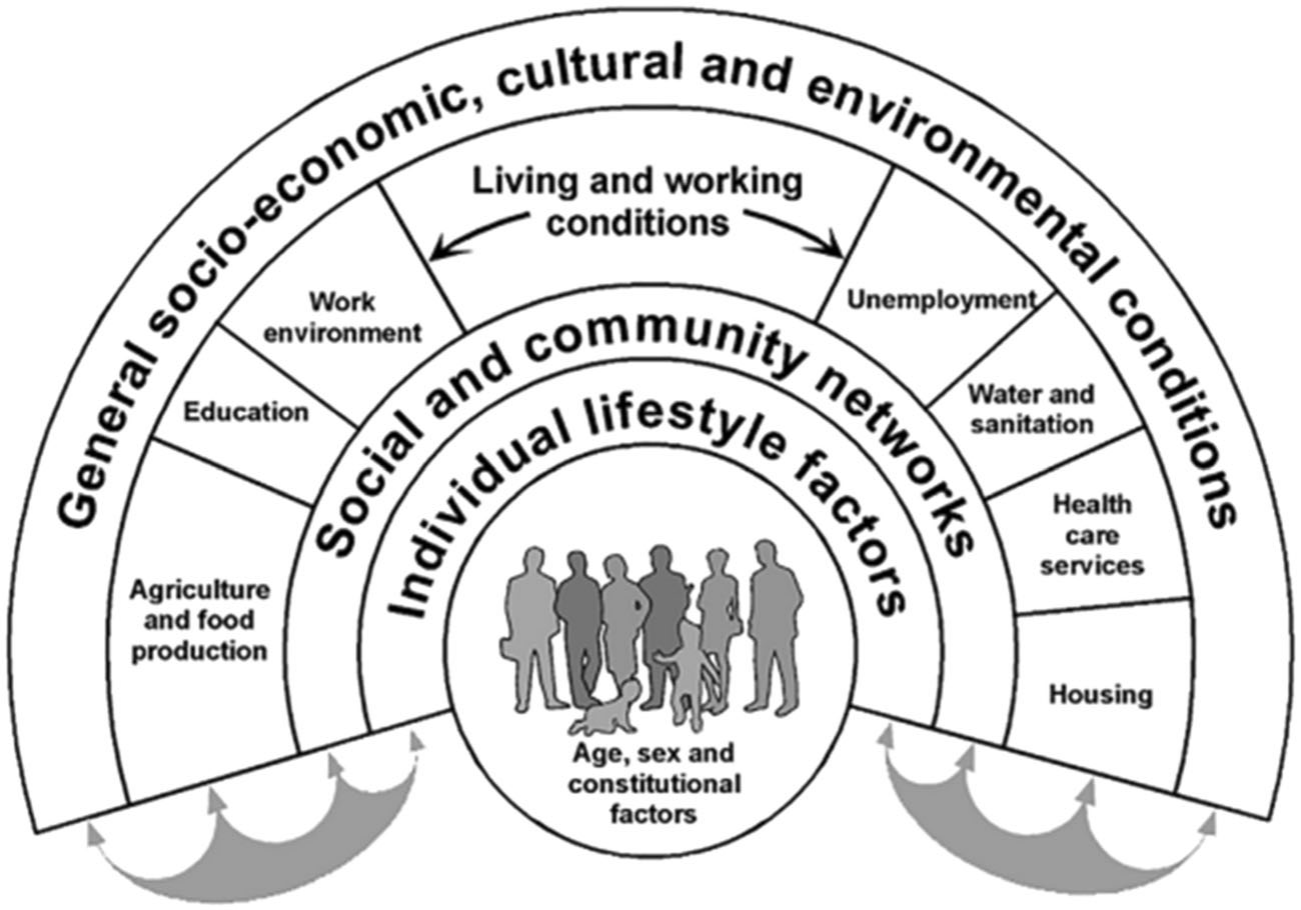
Dahlgren‐Whitehead Rainbow Model (*Source*: Dahlgren & Whitehead^47^).

Health inequality has been a topic of public health interest as far back as 1840.^43^ Over the years, several landmark reports including the Black Report, Acheson Report, and Marmot Review, have established health inequality as a multidimensional problem of the Century^43^, ^44^, ^45^, ^46^ Going further, the causes of health inequalities concerning undernutrition in CUAFY are multidimensional, and for a deeper understanding of these causes, the Dahlgren‐Whitehead Rainbow Model (DRM) (Figure 1) will be used for the explanation of these inequalities.

The DRM was a model developed in 1991 by Göran Dahlgren and Margaret Whitehead.^47^, ^48^ The DRM maps the associations between an individual, his/her immediate and broader environments, and health. In the DRM, individuals are put at the center, and around them are different layers of health influencing factors—including individual lifestyle, societal influences, working and living conditions, and broader social conditions.^49^ However, before proceeding to discuss these health inequalities using the DRM, it is vital to first describe the social gradient of child nutrition and the theories explaining the determinants of nutrition inequalities in children as they form the basis for a deeper and broader understanding of the DRM.^42^ Social gradient in health is a terminology used to describe a concept where people who have more privilege in terms of socioeconomic position/resources (such as education, income, and wealth) have better health conditions and longer lives compared to those who are less privileged.^50^, ^51^ A typical illustration of a social gradient regarding child nutrition was the IDEFICS (Identification and prevention of Dietary‐ and lifestyle‐induced health EFfects In Children and infantS) Study of 8624 children from eight European countries, where significant disparities were observed between sociodemographic variables and body weight.^52^

Several theories have generally explained the determinants of health inequalities. These theories include the social selection theory, materialist (or structural) theory, behavioral theory, psychosocial theory, biological processes theory, and life course theory among others.^42^, ^53^

The social selection theory proposes that people are predisposed to sort themselves into social groups, neighborhoods, and other clusters^54^ (cited in Arcaya et al.^50^). For example, wealthy and highly educated people tend to live in their clusters while poor and lowly educated people tend to live in their clusters. This social segregation explains why child undernutrition is commoner in poorer communities than in wealthier communities.^55^

The materialist theory explains that unequal distribution of socioeconomic resources occurs due to economic structure; in other words, the theory explains the impacts of tangible material resources, including income, on health^56^ (cited in Benzeval et al.^42^). Based on the materialist theory, poverty is a factor that exposes people to health‐related hazards; people with little or no material resources are more likely to live in more hazardous places or places with poor living standards (and social amenities) while people with more material resources are more likely to live in less hazardous places or places with better living standards (and social amenities).^42^ However, many public health experts concluded that the materialist theory is simplistic and insufficient to explain the cause of health inequalities, as some States (including the UK, and the social democratic States ‐ Norway, Sweden, Denmark and other Scandinavian States) provide support (such as school meals, rents, unemployment benefits, etc.) to disadvantaged people in their populations.^53^ Nonetheless the limitations of this theory, research has associated higher rates of undernutrition among children of poor parents/guardians (and in poor communities).^55^

The behavioral theory asserts that a link exists between people's socioeconomic strata and their health which occurs due to differences between socioeconomic strata concerning health‐related behavior.^53^ Health‐damaging behaviors have been linked to human capital^42^; this may explain why unhealthy dietary intake is commoner among children of parents or guardians who had lower human capital than those having parents with higher human capital.^53^, ^57^, ^58^

The psychosocial theory explains the link between social structure (and gradient), and the psychology and health of an individual.^42^, ^53^ According to the theory, social inequality or marginalization causes long‐term feelings of inferiority or subordination in people causing chronic stress that negatively impacts their mental and physical health^53^; this may explain why undernutrition is commoner amongst children from marginalized families/ethnic groups compared to the privileged families/ethnic groups.^59^

The biological processes theory explains that all socioeconomic, psychosocial, and physical environments that can influence health can bring about some biological changes that may cause illness.^42^ According to the theory, people with exposures to higher allostatic load tend to have poorer health indices than those with lower allostatic load^60^; this explains why children with higher allostatic loads (such as chronic hunger, living in unstable homes, and psychosocial deprivation) tend to have higher growth failure rate compared to those children with lower allostatic loads.^61^

Life course theory discusses the multiple factors that shape people's lives from birth to death examining various stages of life. Human life undergoes transition and trajectories with significant implications for human health. These transitions and trajectories represent life phases that are intricately intertwined not only for the individuals but also with the lives of others across the past and future generations. The theory depicts the complexities of lives, by exploring the intricate interrelationship shaped by social structures, time, place, and history.^62^

## 6 SOCIAL DETERMINANTS OF INEQUALITIES OF UNDERNUTRITION IN CUAFY

Having discussed social gradient and the theories explaining the determinants of nutrition inequalities in children, the multi‐level determinants of undernutrition in CUAFY, based on the DRM (Figure 1), can now be discussed further.

### 6.1 Individual level

At the individual level, the determinants of undernutrition amongst CUAFY can be grouped into four thematic areas: biological factors, behavioral factors, psychosocial factors, and physical factors.

Biological factors such as age, sex, and background medical conditions (e.g., genetic and congenital problems) are key determinants of undernutrition in CUAFY.^63^, ^64^, ^65^ Children with genetic and congenital problems, such as congenital heart diseases, orofacial clefts, and Down syndrome, tend to have serious nutritional problems and associated growth failures, unlike healthy children, ceteris paribus.^63^, ^66^, ^67^ Meanwhile, increasing age and increasing sex can either increase the odds of undernutrition amongst CUAFY or vice‐versa.^65^, ^68^ In Ethiopia, for example, male and younger children have higher odds of being undernourished than their counterparts.^65^ On the contrary, in Myanmar, older children and female children have higher odds of being undernourished than their counterparts.^68^ The reasons for these observed intercountry differences in these two individual‐level determinants—age and gender—may be due to the influence of macro (or higher level) determinants such as sociocultural influences, government policies, and so on.^64^, ^69^

It is also noteworthy that behavioral factors such as mental health, choice of food, and feeding patterns are individual‐level determinants of undernutrition in CUAFY.^65^, ^66^, ^67^, ^70^ This explains why children with feeding phobia, selective intake of food, and frequently low appetite for foods are more likely to be undernourished than children with no such problem.^70^

Physical factors such as birth weight and disability are also key individual‐level determinants of undernutrition in CUAFY; children with low birth weight, and disabled children tend to be undernourished compared with children born with higher weight and without disabilities.^65^, ^71^ The aforementioned gives credence to why children affected by severe acute undernutrition commonly have disabilities and higher odds of dying even if they can survive an initial episode of undernutrition.^71^

### 6.2 Working and living conditions, and social influences: Higher level determinants

After individual‐level determinants, working and living conditions, and social influences are the next upper‐level determinants of nutrition status in CUAFY.

Working conditions reflect socioeconomic status. Socioeconomic status—which is an epidemiological variable related to working conditions^72^—is described by the American Psychological Association^73^ as the class or social standing of a person or group. It is usually measured as a combination of occupation, education, and income.^73^ The occupation status and income of the parent(s)/guardian(s) go a long way regarding the nutrition status of CUAFY^74^; CUAFY with poor parent(s)/guardian(s) have higher odds of being undernourished compared to CUAFY with rich parent(s)/guardian(s).^74^, ^75^, ^76^ The level of educational attainment of the parent(s)/guardian(s) is also a key determinant of undernutrition amongst CUAFY; CUAFY with parent(s)/guardian(s) with lower educational qualifications are more likely to be undernourished than those with higher qualifications.^74^, ^77^ The income of a child's parent also goes a long way in determining the likelihood of undernutrition in a child, as children with poorer parents have higher odds of being undernourished.^65^

Living condition is another key determinant of nutrition status in CUAFY. Children living under the care of mentally ill parent(s)/guardian(s), and/or those children with higher birth order have higher odds of being undernourished than those with mentally stable parent(s)/guardian(s) and/or those with lower birth order.^78^, ^79^ Furthermore, the mother's marital status and age are also a set of living conditions associated with undernutrition among CUAFY.^65^ In Ethiopia, for example, CUAFY living with younger mothers and/or mothers with lower body mass index (BMI) have higher odds of being undernourished compared to those with older mothers and/or mothers with higher BMI.^65^

Social influence plays a key role in determining the nutritional status of CUAFY and refers to a change in an individual's emotional state, thoughts, disposition, or behaviors that occur as a result of interaction with another individual or group.^80^ The social influences concerning undernutrition in children include parental and family influence, peer influence, and television (TV) influence.^81^ Parents influence the feeding patterns of their children. While some parent(s)/guardian(s) are responsive, others may be negligent, lazy, or domineering/controlling^70^; and logically, children being fed by responsive parents tend to be more properly fed compared to those that are negligent, lazy, or domineering/controlling, ceteris paribus. Also, parents': food preparation skills, food choices, capability/motivation to help children eat healthy diets, and monitoring of child growth and health influence a child's nutritional status.^81^ Concerning peer influence, peers' eating patterns, choices, and comments can influence eating behaviors in children^81^; invariably, determining the nutritional status of a child in the long term. Concerning TV influence, some parents are being influenced to feed on junk foods through junk food promotions on TV, making them feed their children with unhealthy foods.^81^ This explains why some CUAFY are well‐nourished while some are undernourished.

### 6.3 Broader social conditions: Highest level of determinants

Broader social conditions such as political stability, territorial wealth, law, and policies (LPs) on labor, money, health, research, and so on, are the highest‐level determinants of nutrition in CUAFY.^82^, ^83^

Political stability plays a major role in perpetuating inequalities in child undernutrition. Politically unstable territories, such as territories affected by war, terrorism, civil unrest, etc. commonly experience a higher prevalence of child undernutrition compared to stable territories.^84^, ^85^

Also, laws and policies play a major role in perpetuating inequalities in child undernutrition. Workable and population‐friendly labor laws and policies that promote job security, the creation of gainful employment opportunities, retirement benefits—minimum wage laws and policies that adequately cater for a good standard of healthy living, taxation laws and policies that promote job creation, industrialization and urbanization, and laws and policies which protects employees' job security—logically go a long way in generally improving financial independence and living conditions of people, affecting CUAFY. This explains why some territories (such as the USA, UK, Denmark, and other Scandinavian countries) with better labor laws and policies have lower prevalence rates of undernutrition in CUAFY compared with territories with worse LPs.^36^, ^53^

Monetary laws and policies largely determine the inflation rate of a territory.^86^ People living in territories experiencing high inflation rates are often faced with the problem of reduced purchasing power.^87^ Also, a hike in food prices is an event that accompanies monetary inflation.^87^ This explains why territories experiencing high inflation rates tend to have a higher prevalence rate of undernourished CUAFY compared to those territories experiencing no or little inflation rate.^87^, ^88^

Territorial wealth plays a key role in determining the standard of living of populations occupying a territory (Organization for Economic Co‐operation and Development.^89^ The yardsticks for measuring territorial wealth are natural capital (such as mineral resources, land, timber, etc.), physical capital, and human capital; these capitals determine the wealth per capita of a territory.^90^ People living in territories with higher wealth per capita generally have access to better living conditions, quality healthcare, and food, unlike those living in territories with lower wealth per capita.^89^, ^91^ This is a health inequality gap which further explains why undernutrition is more prevalent in poorer countries.^89^, ^91^

## 7 BRIDGING THE INEQUALITY GAPS IN UNDERNUTRITION IN CUAFY: PUBLIC HEALTH INTERVENTION MODELS

There are two major public health models—the biomedical and the bio‐psychosocial models—that need to be considered concerning the use of public health interventions in eradicating undernutrition in CUAFY.

### 7.1 The biomedical model

The biomedical model, sometimes called the germ theory, explains that a disease occurs due to aberrations from the standard and measurable somatic (biological) variables; the theory provides no accommodation in its framework for the behavioral, psychological, and social aspects of illnesses.^92^ The main focus is on biological factors in disease causality. The model dominates the Western healthcare perspective which has been faulted in many ramifications. The most important shortcoming of the model is that it cannot explain many forms of illness. The model assumes that there is a single underlying cause which is a biological pathology. Restoration of health involves the elimination of the pathology.^93^ Rocca and Anjum^92^ further explained the biomedical model as the foundational notion of diseases which helped to dispel supernatural myths and misconceptions.

More importantly, the model depicts that the origin of disease is a malfunction of the structural and functional level of organisms which is the cell. Hence, the restoration of health also involves pharmaceutical interventions. Going by the definition of health by the WHO, this biomedical analysis focuses on only the pathophysiology, thereby neglecting the socio‐psychological aspects, and is thus, a form of reductionism or physicalism.^92^ The biomedical model fuels medicalization, a situation where life and existential events are defined as mainly biomedical and thus requiring medical interventions.^94^ The model fails to consider the patients as social agents but as biological agents with objectified bodies, which can be manipulated.

### 7.2 The bio‐psychosocial model

The bio‐psychosocial model explains that a disease develops through an intricate interaction between socioenvironmental factors, biology, and psychology.^95^ Unlike the bio‐psychosocial model, the biomedical model is considered “too Western”, and generally associated with greater health inequalities and poorer outcomes.^93^ This model recognizes that not all illnesses are diagnosable through pathophysiological measures. There is a need for holistic measurements which will consider these spheres of life—bio‐psychosocial spheres. This is in line with the WHO's conception of health, which also attracts some criticisms as a vague notion of health with some operational problems.^96^, ^97^ However, the idea of a holistic conception of health is more beneficial and encompassing when building interventions. For instance, a study explained the significance of the interplay among medical, nutrition, feeding skills, and psychosocial characteristics in any intervention to facilitate holistic care of the undernourished.^98^ Hence, nutritional rehabilitation should always consider psychosocial conditions and nutrition as a bidirectional process—psychosocial factors affect nutrition and vice‐versa.

## 8 BRIDGING THE INEQUALITY GAPS IN UNDERNUTRITION IN CUAFY: PUBLIC HEALTH INTERVENTION APPROACHES

Beyond the models, there are three public health intervention approaches in public health: horizontal approach, vertical approach, and diagonal approach. As Table 1 depicts, the vertical approach is a disease‐specific top‐bottom approach that focuses on specialized clinical interventions—diagnosis and treatment.^99^ It is oftentimes a self‐contained health program in the healthcare system, and associated with mass health campaigns targeted at the control or eradication of specific health conditions.^100^ Major examples of vertical approaches translated into public health intervention include the Direct Observed Treatment Strategy (DOTS) for tuberculosis, National Immunization Days (NIDs) aimed at controlling/eradicating polio, and the Onchocerciasis Control Program (OCP) which focused on eliminating onchocerciasis,^101^ as well as HIV/AIDS control programs. Evidence suggests these interventions have produced positive outcomes with low risk to the partakers as both disease conditions have been successfully controlled.^101^

**Table 1:** Features of horizontal, vertical and diagonal health intervention approaches.

**Horizontal approach**	**Vertical approach**	**Diagonal approach**
Addresses overall health problem	Targets specific health problem or population	Targets specific health conditions through health system strengthening
Intervention is long‐term such as establishment of a permanent institution and is usually sustainable	Intervention is short‐term such as through “single‐purpose machinery” and may produce a short‐term result.	Long‐term intervention built into the existing healthcare systems.
Provide promotive, preventive, and curative services	Services aimed at the control or eradication of targeted health concerns or a few diseases.	Combines elements of both vertical and horizontal approaches to address health challenges
Service provided using multi‐purpose resources such as the workforce, finance, etc.	Utilizes resources e.g., finance specifically meant strictly for the targeted disease or health issue.	Combines elements of both vertical and horizontal approaches
Involves the creation of new platforms, e.g., hospital infrastructure, workforces, and other physical or abstract structures thereby impacting the delivery of other interventions	Make use of existing resources	Combines elements of both vertical and horizontal approaches
Objectives are broad in scope and aim to address multiple health issues simultaneously	Have limited objectives focusing on specific illness conditions.	Prioritize interventions aimed at specific health conditions that have a significant impact on public health
Impact on health conditions is indirect	Has a direct impact on health conditions	Maximizes health investments and improves the health outcomes for the population
Financed through government revenues	Often financed by external funders and is time‐bound	Public financed health programs
Targets overall population	Targets disadvantaged populations or groups	Focuses on the population
Integrates completely into the healthcare system as a component of the broader health strategy	Run side‐by‐side and in addition to routine healthcare services	Combines elements of both vertical and horizontal intervention approaches
Comprehensive and holistic approach to health promotion and disease prevention	Approach limited to disease control or eradication	Flexible and adjusts strategies and interventions based on changing epidemiological trends, emerging health threats, and feedback from communities

*Source*: Compiled by the authors.

The horizontal approach is a more encompassing approach toward disease eradication that focuses on the broader determinants of diseases—sociocultural factors like poverty, education, sanitation and nutrition, and so forth,—in a population^97^. These determinants may intersect different sectors and dimensions of public health. Horizontal interventions are focused on the delivery of promotive, preventive, and curative health services by utilizing multipurpose health personnel.^100^ The goal is to make the overall structure and function of the healthcare system,^102^ well‐being and outcomes better. The Expanded Program on Immunization (EPI) has also been effective, especially in polio‐endemic regions.^101^

**Figure 2:**
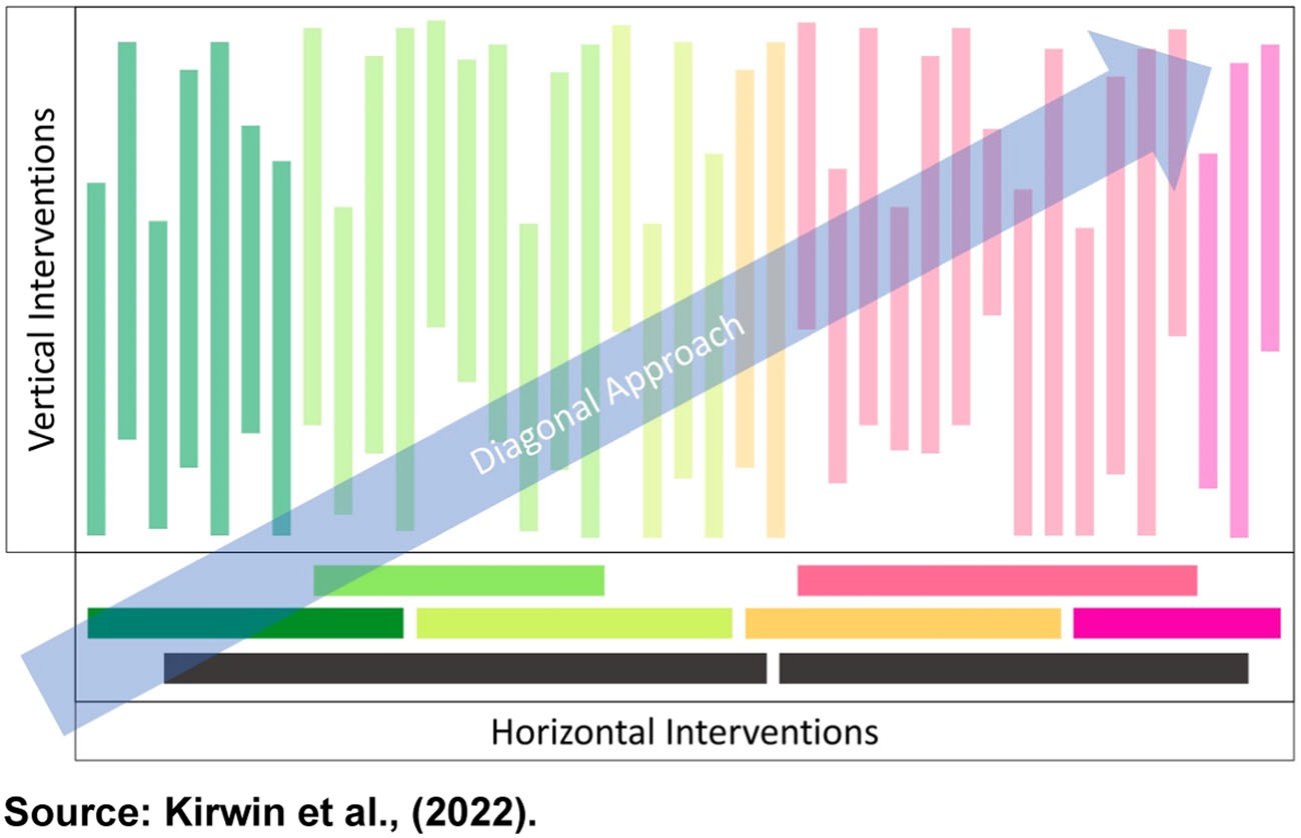
Image depicting diagonal health intervention approach and vertical and horizontal approaches. *Source*: Kirwin et al.^102^

Both approaches have significant limitations, as argued by Béhague and Storeng,^103^ birthing the recommendation of the diagonal approach which is a hybrid of the vertical and horizontal approaches.^102^ Table 1 and Figure 2 provide insights into both the distinctions and connections among the three intervention approaches.

While the horizontal approach has been extolled as a sector‐wide approach which is not disease‐specific like the vertical approach, Kirwin et al. presented the diagonal approach as a mix of the former two approaches as shown in Figure 2. The diagonal approach targets specific diseases combined with health system strengthening. The diagonal approach signifies an optimal combination of horizontal and vertical interventions.^102^ A diagonal approach to scale‐up of primary healthcare systems has been demonstrated in maternal and child health, as a form of integrated and inclusive care.^104^ For instance, targeting various childhood diseases might also incorporate nutrition, sanitation and vaccines which could help fight water‐borne illnesses and vaccine‐preventable diseases. Therefore, a diagonal approach can help improve nutrition and generally reduce child mortality.^105^ A good example of the successful implementation of a diagonal approach in public health is the elimination of measles in the United States and China.^106^

## 9 CONCLUSION

Undernutrition in CAUFY, compounded by its associated health inequalities, represents a significant and multidimensional contemporary public health challenge with long‐term and devastating impacts on global economic, health, and socio‐cultural development. While available data offers insights into the prevalence of stunting and wasting across different regions, it is crucial to acknowledge certain limitations, particularly the lack of information on underweight and vitamin/mineral deficiency. Our review indicates that progress in international efforts and initiatives to eradicate undernutrition has been slow, as the burden continues to disproportionately affect vulnerable populations. The diverse theoretical models discussed in this review underscore the importance of considering the complex interplay of factors at different levels in understanding undernutrition among CUAFY.

This narrative review has provided insights into how the global burden of undernutrition in CUAFY can be addressed through its root causes using a holistic approach that prioritizes equity, promotes social justice, and empowers communities to address their unique health needs. The authors contend that the burdens and inequalities of undernutrition in CUAFY can be reduced, if not eradicated, through comprehensive public health intervention models and approaches that address current challenges concerning child undernutrition. In other words, by implementing targeted interventions that address the social gradient and inequalities perpetuating undernutrition, policymakers and public health practitioners can work toward achieving the goal of eradicating undernutrition globally. However, sustained efforts, investment in research, and collaboration across sectors are essential to making significant strides in reducing the burden of undernutrition and improving the health outcomes of CUAFY worldwide.

Furthermore, public health intervention approaches such as vertical, horizontal, and diagonal approaches offer strategies to tackle undernutrition effectively. While the vertical approach targets specific diseases with clinical interventions, the horizontal approach addresses broader determinants of diseases. The diagonal approach, combining elements of both vertical and horizontal approaches, offers a comprehensive strategy by targeting specific diseases while strengthening health systems. Hence, public health interventions, including horizontal, vertical, and diagonal approaches, must integrate clinical and population‐based strategies to effectively address the multidimensional nature of undernutrition.

## AUTHOR CONTRIBUTIONS

**Kehinde Kazeem Kanmodi**: Conceptualization; investigation; funding acquisition; writing—original draft; methodology; validation; visualization; writing—review and editing; software; formal analysis; project administration; data curation; supervision; resources. **Jimoh Amzat**: Investigation; writing—original draft; writing—review and editing; resources; supervision. **Kafayat Aminu**: Writing—original draft; writing—review and editing; resources.

## CONFLICT OF INTEREST STATEMENT

Kehinde Kazeem Kanmodi is an Editorial Board member of Health Science Reports and a coauthor of this article. To minimize bias, they were excluded from all editorial decision‐making related to the acceptance of this article for publication. The other authors have no conflict of interest to declare.

## TRANSPARENCY STATEMENT

The lead author Kehinde Kazeem Kanmodi affirms that this manuscript is an honest, accurate, and transparent account of the study being reported; that no important aspects of the study have been omitted; and that any discrepancies from the study as planned (and, if relevant, registered) have been explained.

## Data Availability

Data sharing is not applicable to this article as no new data were created or analyzed in this study.
